# Elevated Levels of Endocannabinoids in Chronic Hepatitis C May Modulate Cellular Immune Response and Hepatic Stellate Cell Activation

**DOI:** 10.3390/ijms16047057

**Published:** 2015-03-27

**Authors:** Eleonora Patsenker, Philip Sachse, Andrea Chicca, María Salomé Gachet, Vreni Schneider, Johan Mattsson, Christian Lanz, Mathias Worni, Andrea de Gottardi, Mariam Semmo, Jochen Hampe, Clemens Schafmayer, Rudolf Brenneisen, Jürg Gertsch, Felix Stickel, Nasser Semmo

**Affiliations:** 1 Department of Clinical Research, University of Bern, Bern 3010, Switzerland; E-Mails: sachsephilip@gmail.com (P.S.); vreni.schneider@ikp.unibe.ch (V.S.); felix.stickel@ikp.unibe.ch (F.S.); nasser.semmo@insel.ch (N.S.); 2 Institute of Biochemistry and Molecular Medicine, University of Bern, Bern 3012, Switzerland; E-Mails: andrea.chicca@ibmm.unibe.ch (A.C.); maria.gachet@ibmm.unibe.ch (M.S.G.); juerg.gertsch@ibmm.unibe.ch (J.G.); 3 Department of Clinical Research, Laboratory of Phytopharmacology, Bioanalytics and Pharmacokinetics, University of Bern, Bern 3010, Switzerland; E-Mails: Johan.Mattsson@insel.ch (J.M.); christian.lanz@dkf.unibe.ch (C.L.); rudolf.brenneisen@dkf.unibe.ch (R.B.); 4 Department of Visceral Surgery and Medicine, Inselspital, University of Bern, Bern 3010, Switzerland; E-Mails: christian.lanz@dkf.unibe.ch (M.W.); andrea.degottardi@ikp.unibe.ch (A.G.); 5 Department of Nephrology, Inselspital, University of Bern, Bern 3010, Switzerland; E-Mail: Mariam.Semmo@insel.ch; 6 Department of Medicine II, Division of Gastroenterology, University of Dresden, Dresden 01307, Germany; E-Mail: jochen.hampe@uniklinikum-dresden.de; 7 Department of Visceral Surgery, University of Schleswig-Holstein, Campus Kiel, Kiel 24105, Germany; E-Mail: clemens.schafmayer@uksh-kiel.de

**Keywords:** endocannabinoid system, hepatitis C, peripheral blood mononuclear cells, inflammation, fibrosis, liver

## Abstract

The endocannabinoid (EC) system is implicated in many chronic liver diseases, including hepatitis C viral (HCV) infection. Cannabis consumption is associated with fibrosis progression in patients with chronic hepatitis C (CHC), however, the role of ECs in the development of CHC has never been explored. To study this question, anandamide (AEA) and 2-arachidonoyl glycerol (2-AG) were quantified in samples of HCV patients and healthy controls by gas and liquid chromatography mass spectrometry. Fatty acid amide hydrolase (FAAH) and monoaclyglycerol lipase (MAGL) activity was assessed by [^3^H]AEA and [^3^H]2-AG hydrolysis, respectively. Gene expression and cytokine release were assayed by TaqMan PCR and ELISpot, respectively. AEA and 2-AG levels were increased in plasma of HCV patients, but not in liver tissues. Hepatic FAAH and MAGL activity was not changed. In peripheral blood mononuclear cells (PBMC), ECs inhibited IFN-γ, TNF-α, and IL-2 secretion. Inhibition of IL-2 by endogenous AEA was stronger in PBMC from HCV patients. In hepatocytes, 2-AG induced the expression of IL-6, -17A, -32 and COX-2, and enhanced activation of hepatic stellate cells (HSC) co-cultivated with PBMC from subjects with CHC. In conclusion, ECs are increased in plasma of patients with CHC and might reveal immunosuppressive and profibrogenic effects.

## 1. Introduction

The endocannabinoid system comprises endocannabinoids (ECs), with arachidonoyl ethanolamide (anandamide, AEA) and 2-arachidonoyl glycerol (2-AG) as prominent examples, cannabinoid receptors CB1 and CB2, and EC degradation enzymes fatty acid amide hydrolase (FAAH) and monoacylglycerol lipase (MAGL). AEA and 2-AG are derivatives of arachidonic acid (AA) conjugated with either ethanolamine or glycerol, respectively, and are synthesized on demand from phospholipid precursors residing in the cell membrane in response to a rise of intracellular calcium levels. Inside the cells, AEA is hydrolyzed by FAAH into AA and ethanolamine while 2-AG is hydrolyzed by MAGL into AA and glycerol [1]. The EC system is involved in many physiological and pathophysiological processes, mainly of the CNS, but it also exerts numerous regulatory effects on inflammation, metabolism and vascular tone. Regarding liver diseases, CB1 and CB2 seem to play opposite roles, with CB2 exerting protective effects against the evolution of steatosis, inflammation, and fibrosis [2,3,4], and CB1 promoting these events instead [5]. EC formation is faint in patients without liver disease, whereas in cirrhosis, fatty liver, acute hepatitis or bile duct obstruction, hepatic and plasma levels of AEA and 2-AG are reported to be increased, with AEA being mainly produced by Kupffer cells and lymphocytes and 2-AG derived from hepatic stellate cells (HSC) and hepatocytes [6,7,8,9,10,11]. Multiple effects of ECs were described, including potent anti-inflammatory activity, apoptosis induction, inhibition of cell proliferation, suppression of cytokine production, and induction of T-regulatory cells (Tregs) [1,12,13,14,15,16]. Accordingly, significant suppression of IL-2 production by 2-AG was observed in leukocytes via activation of PPAR-γ [17], whereas the stimulation of CB2 in Kupffer cells prevented the switch to a pro-inflammatory M1 phenotype and enhanced transition towards the anti-inflammatory M2 phenotype [18]. 

Hepatitis C virus (HCV) infection is among the most frequent causes of chronic liver injury leading to cirrhosis. Recent experimental and clinical data in humans demonstrate an association of ECs and cannabinoid receptors with liver disease progression, including chronic hepatitis C (CHC) and non-alcoholic fatty liver disease (NAFLD) [19,20]. Patients with CHC and daily cannabis consumption showed more severe fibrosis than non- or occasional consumers [21,22,23]. *In vitro*, HCV-transfected hepatocytes showed a strong upregulation of CB1 [24]. However, the particular role and effects of ECs in viral-induced liver damage is still incompletely defined. Therefore, we aim to explore the levels of ECs in healthy and diseased cohort of HCV patients and to evaluate whether and how possible alterations could affect immune and fibrogenic responses.

## 2. Results

### 2.1. Endocannabinoid (EC) Levels Are Increased in Plasma of Patients with Chronic Hepatitis C (CHC)

**Figure 1:**
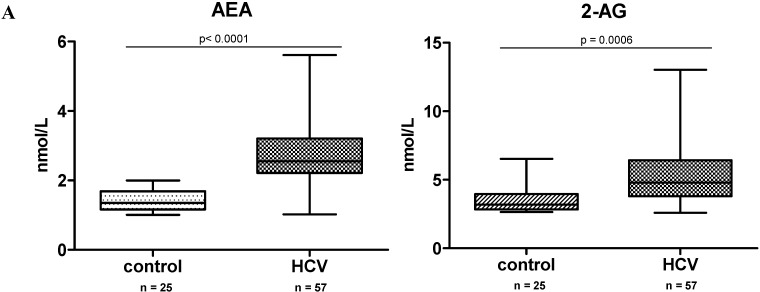

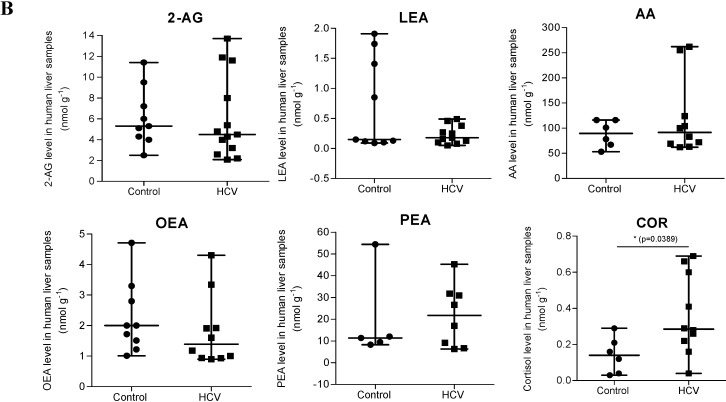
Endocannabinoid (ECs) and related fatty acid amides expression in hepatitis C. (**A**) Levels of ECs in plasma from patients with chronic hepatitis C (CHC) (*n* = 57) and healthy controls (*n* = 25); (**B**) levels of ECs, fatty acids amides and cortisol in control (*n* = 9) and hepatitis C virus (HCV)-infected (*n* = 17) liver tissues. * *p* = 0.0389 *vs.* control.

The levels of ECs AEA and 2-AG were approximately twofold higher in plasma of patients with HCV compared to healthy controls (*p* < 0.005) (Figure 1A), whereas in the liver 2-AG was not changed significantly (Figure 1B) and AEA was below detection limits (not shown). A panel of other related primary fatty acid amides was measured in parallel (linoleoylethanolamide (LEA), palmitoylethanolamide (PEA), oleoylethanolamide (OEA), arachidonic acid (AA)) and, again, no significant differences between HCV and controls groups were observed, except of a stress response marker cortisol, which was induced twofold in the HCV group (Figure 1B).

Interestingly, there was no significant correlation found between plasma EC levels and liver inflammation or fibrosis stages in HCV patients (Table 1).

**Table 1:** Predictors of high endocannabinoids among hepatitis C virus (HCV) patients.

**Anandamide**						
**Parameter**	**Unadjusted Coefficients**	**95% CI**	***p*-Value**	**Adjusted Coefficients**	**95% CI**	***p*-Value**
Age	−0.00066	−0.009–0.007	0.87	−0.0036	0.013–0.0057	0.45
Female	0.075	−0.08–0.23	0.35	0.14	−0.04–0.33	0.13
BMI	0.011	−0.009–0.032	0.27	0.018	−0.0045–0.041	0.12
Diabetes	0.16	−0.12–0.43	0.26	0.14	−0.16–0.45	0.35
Alcohol	0.046	−0.18–0.27	0.69	0.056	−0.21–0.32	0.68
ALT	0.0006	−0.0003–0.002	0.20	0.001	−0.0005–0.0026	0.18
AST	0.00064	−0.0007–0.002	0.35	−0.0003	−0.0025–0.0019	0.81
Fibrosis grade (0–2 *vs.* 3/4)	−0.30	−0.20–0.24	0.73	−0.09	−0.28–0.10	0.36
Fibrosis grade cont.	−0.0034	−0.063–0.056	0.91			
Genotype (1 *vs.* 2–4)	0.087	−0.071–0.25	0.28			
Activity grade (1 *vs.* 2, 1 *vs.* 3)	0.082 −0.27	−0.16–0.33 −0.98–0.43	0.51 0.45			
Steatosis	−0.092	−0.33–0.14	0.44			
2-AG	0.012	−0.069–0.092	0.78			
**2-AG**						
**Parameter**	**Unadjusted Coefficients**	**95% CI**	***p*-Value**	**Adjusted Coefficients**	**95% CI**	***p*-Value**
Age	0.033	0.009–0.058	0.007	0.043	0.014–0.072	0.004
Female	−0.069	−0.59–0.45	0.80	−0.14	−0.72–0.44	0.64
BMI	0.018	−0.049–0.085	0.59	0.029	−0.042–0.10	0.42
Diabetes	−0.15	−1.05–0.75	0.75	−0.54	−1.49–0.41	0.27
Alcohol	−0.33	−1.06–0.40	0.38	−0.07	−0.91–0.77	0.87
ALT	0.003	−0.002–0.007	0.23	−0.00008	−0.005–0.005	0.97
AST	0.002	−0.0009–0.005	0.17	0.0027	−0.004–0.01	0.44
Fibrosis grade (0–2 *vs.* 3/4)	−0.03	−0.58–0.51	0.90	−0.25	−0.85–0.35	0.41
Fibrosis grade cont.	0.009	−0.18–0.20	0.93			
Genotype (1 *vs.* 2–4)	0.087	−0.071–0.25	0.28			
Activity grade (1 *vs.* 2, 1 *vs.* 3)	0.15 −0.43	−0.56–0.86 −2.50–1.63	0.69 0.68			
Steatosis	−0.52	−1.21–0.18	0.14			

Adjustment performed for: age, gender, BMI, diabetes, alcohol, alanine aminotransferase (ALT), aspartate aminotransferase (AST), and fibrosis grade.

### 2.2. Activities of EC Degradation Enzymes Are not Affected in CHC Patients

**Figure 2:**
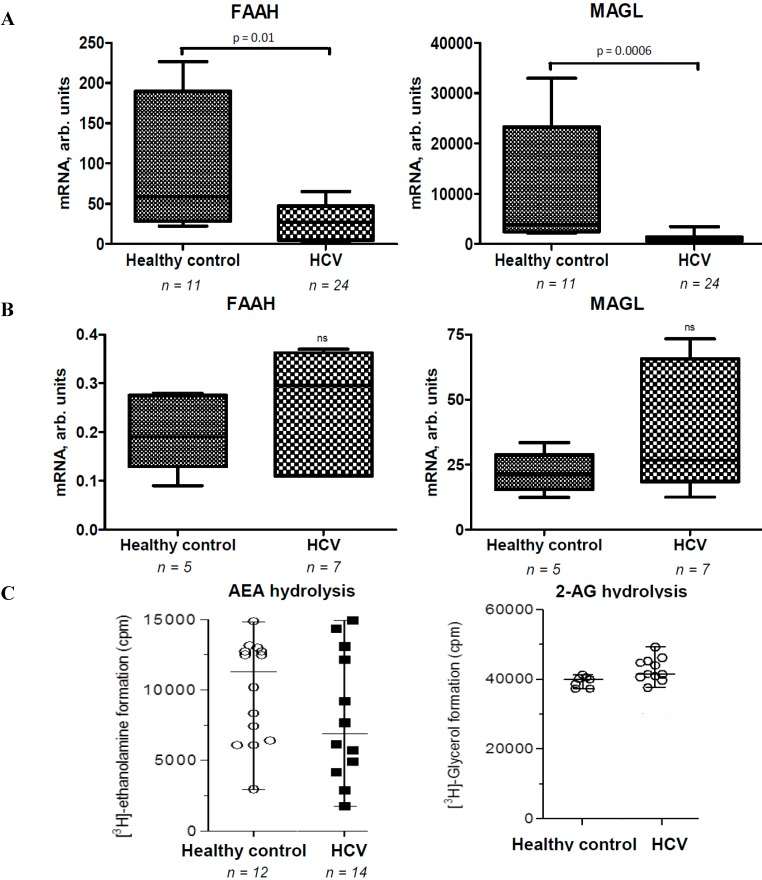
Fatty acid amide hydrolase (FAAH) and monoaclyglycerol lipase (MAGL) mRNA and activity in CHC patients and healthy controls. (**A**) FAAH mRNA and MAGL mRNA in liver biopsies from CHC patients and control livers; (**B**) FAAH mRNA and MAGL mRNA in PBMC from CHC patients and healthy controls; (**C**) FAAH and MAGL enzymatic activity in liver biopsies from CHC patients and control livers. ns—not significant.

The expression of corresponding EC degradation enzymes was measured in parallel in peripheral blood mononuclear cells and liver tissues from healthy controls and HCV patients. Surprisingly, FAAH and MAGL mRNA was not changed significantly in PBMC from HCV-infected patients, but strongly downregulated in liver tissues compared to controls (*p* < 0.05) (Figure 2A,B). In spite of significant mRNA changes, the enzyme activities of FAAH and MAGL in the liver were not altered significantly in the CHC group (Figure 2C).

### 2.3. 2-AG Has a Major Impact on Expression of Components of the EC System in PBMC

**Figure 3:**
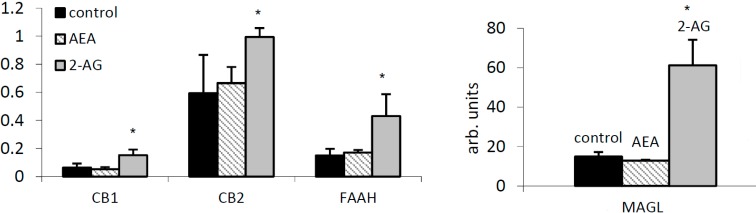
Internal regulation of the EC system by arachidonoyl ethanolamide (AEA) and 2-arachidonoyl glycerol (2-AG). Cannabinoid receptors CB1, CB2, FAAH and MAGL mRNA expression in peripheral blood mononuclear cells (PBMC) by AEA (2.5 µM) and 2-AG (20 µM) was measured by TaqMan PCR and normalized to 18S RNA. * *p* < 0.05 *vs.* untreated control.

The basal gene expression levels for FAAH, MAGL, CB1, and CB2 were measured in peripheral blood mononuclear cells (PBMC) from healthy controls to evaluate the possible mechanisms and regulation. The expression of MAGL was more than 100-fold higher when compared to FAAH mRNA, and CB2 mRNA was 10-fold higher than that for CB1 (Figure 3). Upon treatment with ECs, 20 µM 2-AG caused significant expression of CB1, as well as both degradation enzymes FAAH and MAGL after 24 h of incubation in culture, whereas 2.5 µM AEA was not effective (Figure 3).

### 2.4. ECs Suppress Inflammatory Cytokine Production in PBMC

**Figure 4:**
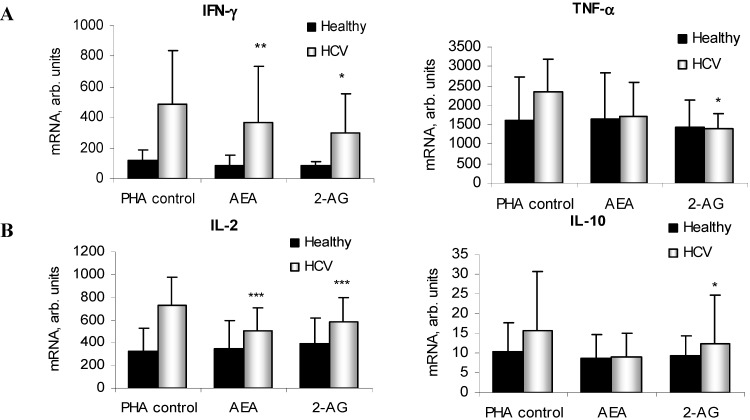

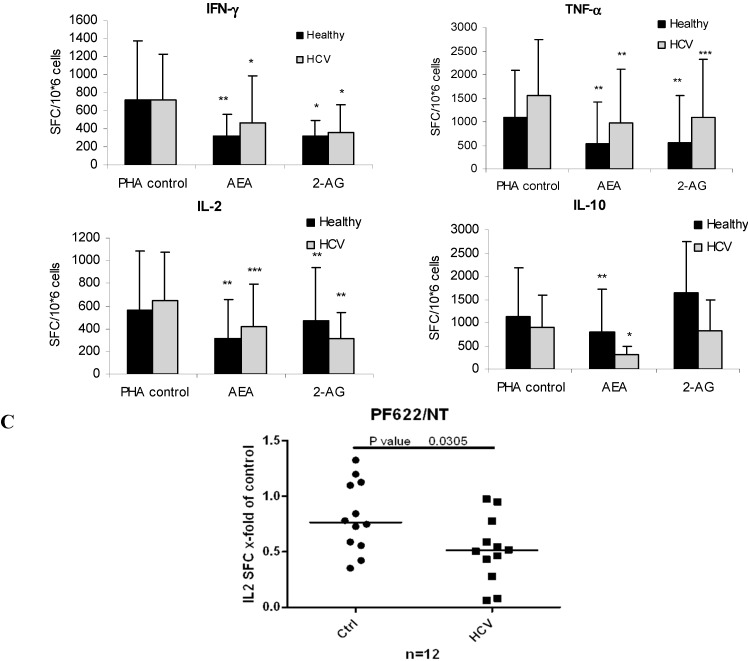
Inflammatory cytokines expression after EC treatment in PBMC. IFN-γ, TNF-α, IL-2 and IL-10 mRNA (**A**) and protein secretion (**B**) in phytohaemagglutinin (PHA)-stimulated PBMC isolated from healthy controls and HCV patients (*n* ≥ 10 each group); (**C**) IL-2 production after FAAH inhibition with PF-622 (1 µM). Results are expressed as arbitrary units or spot-forming cells (SFC) per 1 × 10^6^ cells. * *p* < 0.05; ** *p* < 0.005; *** *p* < 0.0005 *vs*. corresponding controls.

Treatment with AEA and 2-AG led to a downregulation of IFN-γ, TNF-α, I2, and IL-10 mRNA in PBMC from subjects with CHC but not from healthy controls (Figure 4A). At the protein level, ECs significantly reduced the release of all cytokines in both groups, except for IL-10 by 2-AG (Figure 4B). Furthermore, specific inhibition of the AEA degrading enzyme FAAH by PF-622 at 1 µM resulted in a decrease of IL-2 production in both healthy subjects and in HCV patients, and this suppression was much stronger in the latter (Figure 4C). These effects were cell death-independent, as confirmed by FACS analysis (Figure S1).

### 2.5. ECs Reduce Inflammatory Response from HCV-Activated PBMC

**Figure 5:**
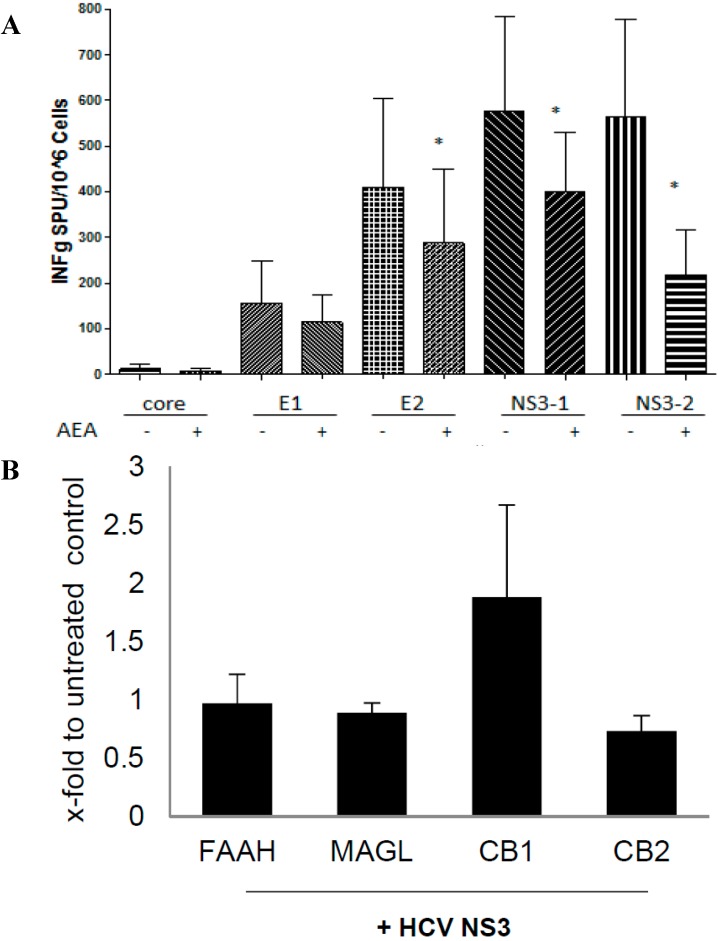
AEA suppresses HCV-induced IFN-γ release in PBMC. (**A**) IFN-γ production in PBMC (*n* = 6) by HCV peptide pools (5 µg/mL) and 2.5 µM AEA. Results are expressed as spot-forming cells (SFC) per 1 × 10^6^ cells; (**B**) FAAH, MAGL, CB1 and CB2 mRNA in PBMC by the NS3 HCV pool. * *p* < 0.05 *vs.* corresponding non-treated control.

Different peptide pools covering the major part of the HCV genome were applied to PBMC isolated from HCV patients. Sufficient induction of IFN-γ was obtained from e1, e2, NS3-1 and NS3-2 pools, which was significantly reduced by AEA at 2.5 µM from 20 to more than 50% in some cases (Figure 5A). Furthermore, NS3 peptides significantly upregulated CB1 mRNA in PBMC, whereas CB2, FAAH and MAGL remained unchanged (Figure 5B).

### 2.6. 2-AG Induces Inflammatory Cytokine Expression in Primary Hepatocytes and Fibrogenic Activation of HSC

**Figure 6:**
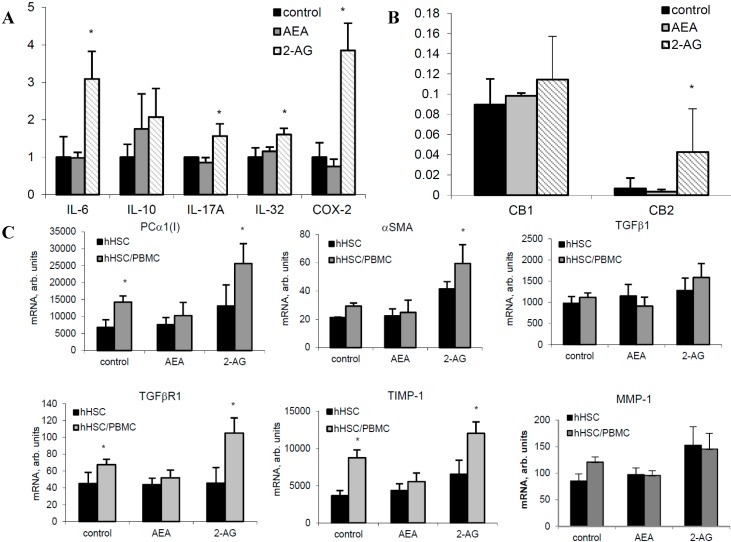
ECs affect inflammatory and fibrogenic hepatic cells activities. (**A**) Inflammatory cytokines mRNA and (**B**) CB1 and CB2 mRNA in human hepatocytes by 2.5 µM AEA and 20 µM 2-AG; (**C**) Fibrosis-related mRNA in hHSC and hHSC co-cultivated with PBMC from CHC patients by 2.5 µM AEA and 20 µM 2-AG. * *p* < 0.05 *vs* corresponding non-treated control.

In hHep, 2-AG induced the expression of IL-6, IL-10, IL-17A, IL-32, and COX-2 mRNA, whereas no significant changes occurred after AEA treatment (Figure 6A). TNFα and TGFβ1 mRNA were not changed by the EC treatment and IFN-γ and IL-2 mRNA were not detectable in primary hHep (not shown). Notably, here the expression of CB1 mRNA was much higher than of CB2, opposing to that of the CB receptors’ expression in PBMC (Figure 6B). Stimulation with 2-AG led to further upregulation of CB2 mRNA by fivefold (*p* < 0.05) but not of CB1 mRNA (Figure 6B).

Co-cultivation of primary hHSC with PHA-stimulated PBMC revealed a strong induction of PCα1(I), TGFβR1 and TIMP-1 mRNA in hHSC (Figure 6C). 2-AG further upregulated PCα1(I), αSMA, TGFβR1, and TIMP-1 mRNA, whereas TGFβ1 and MMP-1 remained non-significantly changed (Figure 6C).

## 3. Discussion

The involvement of CB1 and CB2 in modulating liver fibrosis, steatosis and cell regeneration has been repeatedly demonstrated [3,25,26]; however, the particular role of AEA and 2-AG, as well as EC degrading enzymes FAAH and MAGL in the development of CHC is not well known. Few studies showed that regular cannabis consumption may promote liver fibrosis and steatosis in CHC patients [21,22,23], but the involvement of EC system in HCV-induced injury has not been well described.

In the present study we show that AEA and 2-AG are increased in plasma from CHC patients compared to healthy controls. In the liver, AEA levels were below detection limits, whereas 2-AG were not different between groups. Clugston *et al.* [27] reported a concomitant reduction of hepatic FAAH expression paralleled by an increase of AEA but not of 2-AG in livers of alcohol-fed mice. Therefore, we hypothesized that in our study the increase of EC levels might be explained by reduced EC degradation enzymes activity. But in spite of a strong downregulation of FAAH and MAGL mRNA expression in the liver, the activity of corresponding enzymes was not changed. Comparable FAAH and MAGL activities between groups may be explained by the large interindividual variability, preventing significance due to the relatively low numbers of patients. Thus, an increase of ECs in plasma could be explained by enhanced EC synthesis in the liver or immune cells or reduced FAAH and MAGL activities from immune cells due to the stress from inflammation or tissue injury, as previously suggested by others [3,28,29,30].

As reported previously, the EC system can modulate inflammatory and immune responses [1,3,15,16,29]; however, its effects on HCV-specific immune reactions has not been delineated. Here we show that ECs suppress inflammatory cytokine secretion in PBMC from non-infected and HCV-infected patients. Accordingly, a rise of the endogenously produced AEA in PBMC induced by selective FAAH inhibition reduced the release of IL-2 to a much higher extent in HCV samples, thus potentially compromising the immune response.

To elucidate whether HCV directly affects the EC system, we applied various HCV peptides to PBMC and found that it markedly induced CB1 mRNA expression. It has been demonstrated that HCV stimulates CB1 and 2-AG production in hepatocytes, resulting in a changed expression profile of glucose metabolism-related genes and increased steatosis [24,31] and, most interestingly, CB1 antagonists inhibit hepatitis C virus production [32] but whether the increase of CB1 impairs PBMC functions still remains to be investigated.

Next, we explored the effects of ECs on inflammation- and fibrosis-related genes in hepatocytes and hHSC, in an attempt to extend our previous work showing 2-AG as an inducer of CB1 and αSMA in hHSC [33]. Although we were not able to detect a significant difference in 2-AG expression in the livers of HCV-infected patients here, it may still be possible that ECs contribute to ongoing liver damage via activated and sensitized liver cells. Thus, in primary hepatocytes, and opposite to that in PBMC, 2-AG strongly upregulated the expression of IL-6, -10, -17A, -32, and, especially, of COX-2 mRNA, the converting enzyme for the main precursor of ECs, arachidonic acid. This observation suggests differential and site-specific regulation of EC system, with higher CB1 expression on hepatocytes, rendering them more susceptible to the pro-inflammatory effects of ECs, and higher CB2 expression on PBMC, making them amenable to the immunosuppressive effects from ECs. Even though CB2 was likewise induced to some extent by 2-AG in hepatocytes, this effect did not seem to be sufficient to overcome the CB1-mediated induction of inflammatory genes. However, this will unlikely lead to increased hepatic inflammation in general, as hepatocytes are not the major contributors to the immune response, and ECs-induced immunomodulatory effects are mostly mediated via CB2 on locally residing Kupffer cells or hepatic or peripheral T-lymphocytes [12,18,34]. This highlights a careful interpretation of the results from *in vitro* studies performed in single cell types before translating obtained findings to the situation in humans.

To explore this further, we studied the impact of ECs on hepatic fibrogenesis by performing a co-cultivation experiment of hHSC with PBMC from HCV-infected subjects. Co-cultivation of PBMC with hHSC *per se* induced a pro-fibrogenic phenotype of hHSC via upregulation of key fibrosis genes PCα1(I), TGFβR1 and TIMP-1 mRNA, and 2-AG further enhanced this effect strongly. Nattermann and colleagues convincingly demonstrated that the ineffective immune response during HCV infection may be directly linked to aggravation of hepatic fibrogenesis, as CD4(+) T cells were shown to stimulate anti-fibrotic NK cell activity via IL-2 mediated upregulation of NKG2D, and an impaired activity of CD4(+) T cells contribute to acceleration of liver fibrosis [35].

Thus, the question of whether increased ECs in CHC promote liver disease progression due to impaired systemic immune response and stimulated fibrogenesis, or alleviate these processes via CB2 activation still remains an open question, as effects are at least in part counteractive [36,37]. Immunosuppressive effects of tetrahydrocannabinol and other cannabinoids have been shown in B-, T-, NK-cells, and macrophages via CB1 and CB2-dependent and -independent mechanisms [15,16], whereas in the liver, direct favorable effects of ECs on inflammation and oxidative stress have been shown in ischemia reperfusion experimental models in rodents [38].

In conclusion, we found that AEA and 2-AG are increased in patients with CHC, and that HCV may directly affect the EC system via regulation of CB receptor expression. Increased EC levels during HCV infection might suppress inflammatory cytokines production, thereby weakening the immune response towards HCV infection. In addition, ECs may aggravate liver fibrosis via direct HSC activation. Whether the EC system halts or perpetuates chronic liver damage, and can thus be a therapeutic target in chronic HCV infection, still remains to be elucidated in more details *in vivo* to account for various confounding factors such as disease stage, viral load, immune status, presence of fibrosis or degree of hepatic inflammation, steatosis, and probably others, calling for a note of caution in targeting the EC system in the treatment of CHC [37].

## 4. Material and Methods

### 4.1. Human Tissues and Patients’ Characteristics

EC levels and EC degradation enzymes activity were measured in liver tissues and plasma samples from patients with CHC and from healthy controls. Plasma samples were obtained from HCV patients (*n* = 57) (Inselspital, Bern) and healthy volunteers (*n* = 25) according to a strict standard operating procedure (SOP) (Supporting Information). Snap-frozen liver tissues were obtained from consecutive HCV patients (Inselspital Bern, Switzerland) who underwent percutaneous liver biopsies for diagnostic reasons (*n* = 17). All biopsies were evaluated for fibrosis and inflammatory activity according to the standard METAVIR scoring system. Histologically normal liver biopsies from patients who underwent bariatric surgery (Department of Visceral Surgery, University of Schleswig-Holstein, Kiel, Germany) (*n* = 6) [39] and cholecystectomy (Inselspital, Bern) (*n* = 3) served as controls. The human study was approved by the ethical committees of the two participating centers. All patients and volunteers gave written informed consent to have their biological materials assayed in a retrospective study. Patients’ characteristics are summarized in Table 2 and Table 3.

**Table 2:** Patients’ characteristics (plasma samples).

	Control	HCV
Number of patients	25	57
Female (n, %)	15 (60)	22 (38.6)
Age (mean, SD)	44.2 (11.4)	52.7 (10.0)
Diabetes (n, %)	0	5 (8.8)
BMI (mean, SD)	NA	24.7 (3.8)
ALT (mean, SD)	NA	115.8 (84.4)
AST (mean, SD)	NA	91.1 (58.8)
INR (mean, SD)	NA	NA
Albumin (mean, SD)	NA	NA
CHILD		
A		14 (24.6)
B		2 (3.5)
C		0
Unknown/NA	25 (100.0)	41 (71.9)
Fibrosis stage		
0		4 (7.0)
1		21 (36.8)
2		11 (19.3)
3		5 (8.8)
4		15 (26.3)
Unknown/NA	25 (100.0)	1 (1.8)
Activity grade		
		19 (33.3)
		14 (24.6)
		1 (1.8)
Unknown/NA	25 (100.0)	23 (40.4)
Steatosis		
No		15 (26.3)
Yes		16 (28.1)
Unknown	25 (100.0)	26 (45.6)
Genotype		
1		2 (3.5)
1A		11 (19.3)
1B		18 (31.6)
2		8 (14.0)
3		14 (24.6)
4		3 (5.3)
Negative		1 (1.8)
Unknown/NA	100 (100.0)	0 (0.0)
Viral load (mean/SD) Missing	NA	2,182,222 (2,705,243) 1 (1.8)

**Table 3:** Patients’ characteristics (liver biopsies).

	Control (Bariatric Surgery)	HCV
Number of patients	6	17
Female (n, %)	4 (66)	6 (35.3)
Age (mean, SD)	34 (8.4)	57.5 (12.7)
Diabetes (n, %)	6 (100)	NA
BMI (mean, SD)	49.5 (7.3)	NA
ALT (mean, SD)	NA	NA
AST (mean, SD)	NA	NA
INR (mean, SD)	NA	NA
Albumin (mean, SD)	NA	NA
CHILD		
A		5 (29.4)
B		0 (0.0)
C		0 (0.0)
Unknown/NA		
Fibrosis stage		
0	6 (100.0)	1 (5.9)
1		0 (0.0)
2		3 (17.6)
3		8 (47)
4		5 (29.4)
Unknown/NA		
Activity grade		
1		6 (35.3)
2		10 (58.8)
3		1 (5.9)
Unknown/NA		
Steatosis		
No	6 (100.0)	5 (29.4)
Yes		12 (70.5)
Unknown		
Genotype	6 (100.0)	
1		
1A		12 (70.5)
1B		NA
2		NA
3		1 (5.9)
4		2 (11.7)
Negative Unknown/NA	6 (100.0)	2 (11.7)
Viral load (mean/SD)	NA	2,530,938 (3,483,784)

### 4.2. Materials

Human peripheral blood mononuclear cells (PBMC) were obtained from whole blood via density-gradient centrifugation as previously described [40]. Primary human hepatic stellate cells (hHSC) and primary human hepatocytes (hHep) were obtained from ScienCell^TM^ Research Laboratories (Carlsbad, CA, USA). AEA and 2-AG were obtained from Sigma-Aldrich, Germany; PF-622, URB-597 and JZL184 from Cayman Chemical, USA; HCV peptide pools from BEI Resources, Manassas, VA, USA. Overlapping peptides derived from the HCV strain H77 (genotype 1a) spanning the complete HCV polyprotein (18 amino acids each, overlapping by 11 amino acids) were used at a final concentration of 5 μg/mL [34]. The composition of the peptide pools is provided in Supporting Information (Table S1).

### 4.3. Analysis of ECs in Human Plasma Samples and Liver Biopsies

Plasma levels of ECs were measured by GC-MS, as will be published in detail elsewhere, while in the liver, ECs were measured by LC-MS, as described in Supporting Information (Table S2).

#### 4.3.1. FAAH and MAGL Activity Assay

Enzyme activities were measured as described previously [41]. Briefly, 1 µM of URB597, JZL-184 or vehicle was pre-incubated for 30 min at 37 °C with 490 μL of diluted liver tissue homogenates (1 mg total protein/sample) in 10 mM Tris-HCl, 1mM EDTA (pH8.0). A mixture of 100 nM AEA plus 1 nM [^3^H]-AEA or 1 µM 2-AG plus 1nM [^3^H]-2-AG was added and incubated for 15 min at 37 °C. The reaction was stopped by transferring the samples to a new tube containing 1 mL of CHCl_3_/CH_3_OH mixture (1:1, *v*/*v*). The solution was vigorously vortexed and then centrifuged at 4 °C for 10 min at 10,000 rpm to separate aqueous and organic phase. The amount of [^3^H]ethanolamine (AEA degradation) or [^3^H]glycerol (2-AG degradation) formed upon enzymatic hydrolysis was quantified by adding 3 mL of Ultima Gold scintillation liquid (PerkinElmer Life Sciences, Long Island, NY, USA) to the aqueous phases and measuring the radioactivity with the Packard Tri-Carb 2100 TR β-counter.

#### 4.3.2. *In Vitro* Experiments

PBMC, hHSC and hHep were cultured in Roswell Park Memorial Institute medium (RPMI), Dulbecco’s modified Eagle's medium (DMEM) or hepatocyte medium (HM; ScienCell, Carlsbad, CA, USA), respectively, containing 10% FBS, 2 mM glutamine, 100 U/mL penicillin and 100 U/mL streptomycin. Cells were maintained at 37 °C in 5% CO_2_ humidified atmosphere. 

hHSC and hHep were seeded onto 12-well plates and, after reaching semi-confluent state, starved in serum-free medium for next 24 h. Treatment with 2.5 µM AEA and 20 µM 2-AG, 1 µM PF-622, 5 µg/mL phytohemagglutinin (PHA) or 5 μg/mL HCV peptides pools was performed for 6–24 h in complete medium containing 0.1% FBS.

Co-cultivation of hHSC and PBMC was performed using 0.4 µm polycarbonate membrane 12 mm inserts (Corning Incorporated, Lifesciences, Lausen, Switzerland).

#### 4.3.3. Quantitative Real-Time PCR

Total RNA was isolated from snap frozen tissues or cultured cells using the RNeasy kit (Qiagen, Basel, Switzerland) and 1 µg transcribed to cDNA as described [42]. Quantitative real-time PCR was performed on the ABI 7700 Sequence Detector (Applied Biosystems, Rotkreuz, Switzerland) using ready-to-use primers and probes sets (Applied Biosystems, Rotkreuz, Switzerland). For normalization, the housekeeping gene 18SRNA was amplified in a parallel reaction.

#### 4.3.4. ELISpot

Cytokine production was determined by ELISpot assay (MABTECH, Stockholm, Sweden) according to the manufacturer’s instructions as previously described [34,40,43,44]. Briefly, PBMC were seeded at 1 × 10^6^ cells/mL and pre-incubated in RPMI with or without ECs for 3 h at 37 °C before stimulation with PHA or HCV peptides, where applicable. The vehicle alone was used as a negative control.

### 4.4. Statistical Analysis

Statistical analyses were performed using Microsoft Excel and GraphPad Prism software. Data are expressed as means ± SD. The statistical significance of differences was evaluated using paired or unpaired Student’s *t*-test or nonparametric ANOVA Kruskal-Wallis test. Predictions were performed using multiple regression analysis.

## Supplementary Materials

Supplementary materials can be found at http://www.mdpi.com/1422-0067/16/04/7057/s1.

## Abbreviations

AEA: anandamide
2-AG: 2-arachidonoyl glycerol
CHC: chronic hepatitis C
FAAH: fatty acid amid hydrolase
MAGL: monoacylglycerol lipase
hHep: human hepatocytes
hHSC: human hepatic stellate cells
