# Supplementary material for: Elevated Levels of Endocannabinoids in Chronic Hepatitis C May Modulate Cellular Immune Response and Hepatic Stellate Cell Activation
Source: Int J Mol Sci. 2015 Mar 27;16(4):7057–76. doi: 10.3390/ijms16047057 (PMC4425004; doi:10.3390/ijms16047057)
Supplement: Supplementary file 1 [file ijms-16-07057-s001.pdf]

Supplementary Information

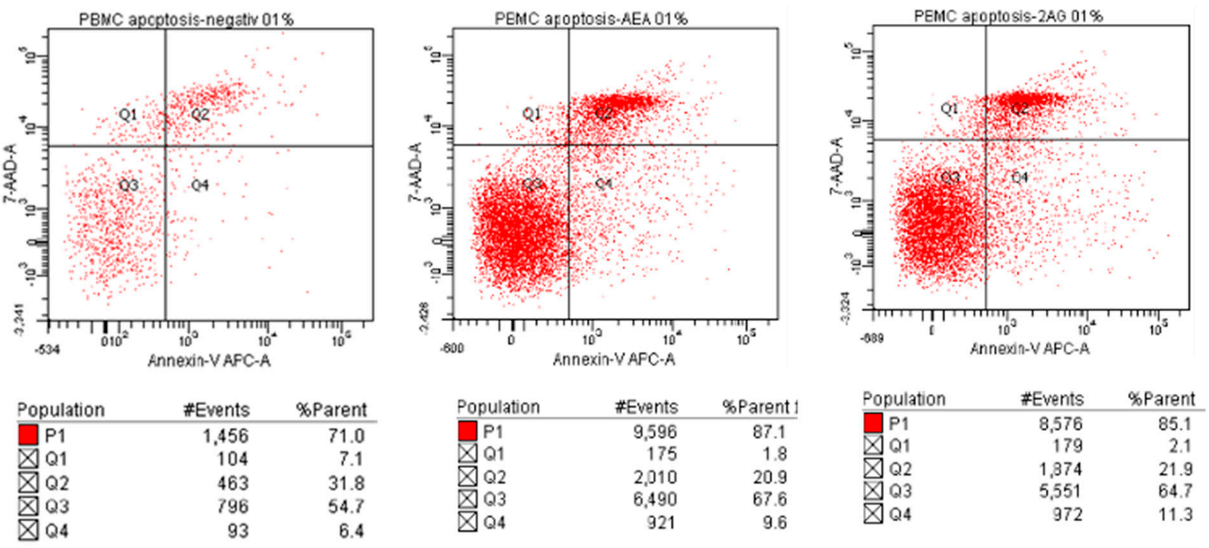

Figure S1. Flow cytometry with Annexin V and 7-AAD for cell death analysis of PBMC treated with 2.5 µM AEA and 20 µM 2-AG. No cell necrosis or apoptosis is observed after 24 h of incubation with endocannabinoids.

Phlebotomy and Preparation of Plasma Samples (Standard Operating Procedure, SOP)

Two fasting blood samples were collected from each subject by venipuncture of the median cubital vein and collected into EDTA plasma tubes (Sarstedt, Sevelen, Switzerland). One of the samples served as a backup sample. The samples were immediately placed on ice after careful mixing. Centrifugation was performed at 4 °C and 2000× *g* for 10 min to obtain the plasma. The plasma was transferred to 5 mL polypropylene screw top vials (Sarstedt) and divided into aliquots (2.5 mL). After snap-freezing in liquid nitrogen, the samples were stored at −80 °C until extraction.

Analytical Quantification of Endocannabinoid and Related Lipid Levels by Liquid Chromatography Tandem Mass Spectrometry (LC/MS)

(a) Chemicals and Reagents:

All chemicals and reagents were of the purest analytical grade. AEA or (5*Z*,8*Z*,11*Z*,14*Z*)-eicosatetraenamide), AEA-*d*4 or *N*-(2-hydroxyethyl-1,1,2,2-*d*4)-(5*Z*,8*Z*,11*Z*,14*Z*)-eicosatetraenamide, 2-AG or (5*Z*,8*Z*,11*Z*,14*Z*)-eicosatetraenoic acid, 2-glyceryl ester, 2-AG-*d*5 or (5*Z*,8*Z*,11*Z*,14*Z*)-eicosatetraenoic acid, 2-glyceryl-1,1,2,3,3-*d*5 ester, palmitoylethanolamide (PEA) or *N*-(2-hydroxyethyl)-hexadecanamide, PEA-*d*5 or *N*-(2-hydroxyethyl)-hexadecanamide-15,15,16,16,16-*d*4, stearoyl ethanolamide (SEA) or *N*-(2-hydroxyethyl)-octadecanamide, oleoylethanlonamide (OEA) or *N*-(2-hydroxyethyl)-9*Z*-octadecenamide, OEA-*d*4 or *N*-(2-hydroxyethyl-1,1,2,2-*d*4)-9*Z*-octadecenamide, linoleoylethanolamide (LEA) or *N*-(2-hydroxyethyl)-9*Z*,12*Z*-octadecadienamide, LEA-*d*4 or *N*-(2-hydroxyethyl-1,1,2,2-*d*4)-9*Z*,12*Z*-octadecadienamide, arachidonic acid (AA) or (5*Z*,8*Z*,11*Z*,14*Z*)-eicosatetraenoic acid and AA-*d*8 or (5*Z*,8*Z*,11*Z*,14*Z*)-eicosatetraenoic-5,6,8,9,11,12,14,15-*d*8 acid, were purchased from Cayman Chemical Europe, Tallinn, Estonia. Hydrocortisone (Cortisol) was purchase from Sigma-Aldrich, Steinheim, Germany. Cortisol-*d*4 was purchase from Alsachim, Illkirch

Graffenstaden, France. HPLC-grade methanol (CH3OH), HPLC-grade acetonitrile, ammonium acetate, formic acid and phosphate buffered saline (PBS) were obtained from Sigma-Aldrich, Steinheim, Germany. HPLC-grade chloroform ($CHCl_3$) was obtained from Biosolve, Valkenwaard, Netherlands. HPLC-grade ethyl acetate was obtained from Acros Organics, New Jersey, USA. Hydrochloric acid 37% and acetic acid were purchased from Merck KGaA, Darmstadt, Germany. Deionized water (18.2 MΩ × cm) was obtained from an ELGA Purelab Ultra Genetic system (VWS (UK) Ltd, ELGA LabWater, UK).

(b) Chromatographic and Mass Spectrometric Conditions for the Quantification of Endocannabinoids and Related Lipids:

Analyses were conducted on an LC/MS/MS system consisting of an API 4000 QTrap mass spectrometer equipped with a TurboIonSpray probe (AB Sciex, Concord, ON, Canada) connected to a Shimadzu UFLC (Shimadzu Corporation, Kyoto, Japan). Data acquisition and analysis were performed using Analyst software version 1.5.1 (AB Sciex, Concord, ON, Canada). Analytical LC separations were performed on a Reprosil-PUR C18 column (3 µm particle size; 2 × 50 mm, A. Maisch, High Performance LC-GMBH, Ammerbuch, Germany) at a flow of 0.35 mL/min and 40 °C using a gradient of methanol containing 2 mM ammonium acetate (solvent B) in water containing 2 mM ammonium acetate and 0.1% formic acid (solvent A) (*i.e.*, 15% of solvent B for 0.5 min, in 3 min to 70%, in 4.5 min to 99% and kept at 99% for 3 min, in 0.5 min back to 15% methanol and conditioned for 1.5 min at 15% of solvent B). Autosampler was kept at 4 °C. The mass spectrum (MS) detection was performed using the TurboIon Spray interface which was operated in negative ionization mode for the analysis of AA and in positive mode for the analysis of AEA, 2-AG, PEA, SEA, OEA, LEA and Cortisol. The parameters of the source using nitrogen as curtain gas were the following: capillary ion spray voltage +4500 V in positive and −4250 V in negative modes, respectively, temperature 600 °C, curtain gas 25 psi, GS1 50 psi and GS2 50 psi. The entrance potential and collision cell exit potential were set to 10 V, respectively. The analytes were measured in multiple reaction monitoring mode (MRM). Two MRMs, one qualifier and one quantifier were considered in the analysis. The MRM parameters (retention time, precursor ion/product ion, declustering potential, collision energy) for the quantifier MRM used in the survey and the respective internal standard (IS) were AA: 8.48 min, 303/59 *m*/*z*, −80 eV, −37 eV; AA-d8: 8.45 min, 311/59 *m*/*z*, −90 eV, −38 eV; AEA: 7.94 min, 348/62 *m*/*z*, 56 eV, 42 eV; AEA-d_4: 7.93 min, 352/66 *m*/*z*, 60 eV, 35 eV; 2-AG: 8.04 min, 379/203 *m*/*z*, 82 eV, 25 eV; 2-AG-d5: 8.02 min, 384/287 *m*/*z*, 62 eV, 17 eV; PEA: 8.13 min, 300/62 *m*/*z*, 78 eV, 36 eV; PEA-d5: 8.12 min, 305/62 *m*/*z*, 70 eV, 40 eV; SEA: 8.64 min, 328/62 *m*/*z*, 72 eV, 31 eV; OEA: 8.31 min, 326/62 *m*/*z*, 72 eV, 36 eV; OEA-d_4: 8.31 min, 330/66 *m*/*z*, 72 eV, 32 eV; LEA: 7.90 min, 324/62 *m*/*z*, 74 eV, 36 eV; LEA-d_4: 7.90 min, 328/66 *m*/*z*, 70 eV, 35 eV; 4.85 min, 367/121 *m*/*z*, 78 eV, 35 eV; Cortisol: 4.86 min, 363/121 *m*/*z*, 76 eV, 38 eV; and Cortisol-d_4: 4.85 min, 367/121 *m*/*z*, 78 eV, 35 eV, respectively. Data were acquired and processed by Analyst software.

Sample Preparation:

Upon collection, liver biopsies were immediately placed in liquid nitrogen and stored at −80 °C until analysis. Lipid extraction was performed according to [1] with slight modifications. In brief, liver tissue

(5–12 mg) was homogenized on a BeadBeater (Mini-BeadBeater-24, BioSpec, Bartlesville, OK, USA) using 2 mL vials and Chrome-Steel Beats (2.3 mm dia, BioSpec) with 1 mL of a mixture of ice-cold $CHCl_3/CH_3OH$ 2:1 (1 min, 3450 strokes/min) at 4 °C. The homogenized samples were placed on centrifuge tubes containing 8 mL of ice-cold $CHCl_3/CH_3OH$ mixture 2:1 (*v*/*v*) and internal standards (10 µL of IS solution prepared in ethanol containing: 200 ng/mL AEA-d_4, PEA-d_5, OEA-d_4, LEA-d_4, 500 ng/mL 2-AG-d_5, 1500 ng/mL Cortisol-d_4 and 15,000 ng/mL AA-d_8). The samples were rinse twice with 1 mL of the $CHCl_3/CH_3OH$ mixture. Finally, 1.5 mL of PBS was added to yield the desired 6:3:1.5 ratio ($CHCl_3/CH_3OH$/PBS, *v*/*v*/*v*). The suspension was vortex vigorously, sonicated for 5 min and then centrifuged for 5 min at 800× *g* at 4 °C. The organic phase was recovered on silanized glass tubes and dried under nitrogen. Subsequently, the samples were reconstituted in 1 mL ethanol, diluted with 9 mL water, adjusted to pH 3 by adding 150 µL hydrochloric acid (0.1 M) and extracted by solid-phase extraction (C-18 Sep-Pak cartridge (Waters AG) pre-activated with 3 mL CH_3OH and equilibrated with 3 mL 10% ethanol). Cartridges were washed with 3 mL 10% ethanol and eluted with 3 mL acetonitrile/ethyl acetate (1:1). The eluates were evaporated to dryness under nitrogen. The samples were reconstituted in acenonitrile to a final volume of 0.1 mL and centrifuged for 5 min at 16,100× *g* at 4 °C. Carefully, 80 µL were pipetted out, placed into conic amber vials and analyzed by LC/MS/MS (10 µL injection volume).

(a) Quantification of Analyte Levels:

In order to quantify the amount of endocannabinoids and related lipids in liver, external calibrations were performed in triplicate. For all the analytes, R^2 values were higher than 0.98 except for PEA (0.94). The recovery of control rat liver samples run together with the human samples was in the range of 80%–105% for all analytes, except for PEA that showed a recovery of 70%. The human liver samples were quantified with calibration curves prepared on rat liver homogenate (CH_3OH/PBS) covering a linear analytical range of 0.16–6.40 ng/mL for AEA and LEA; 0.16–40 ng/mL for OEA; 6.4–100 ng/mL for PEA; 0.33–12.80 ng/mL for Cortisol; and 1.64–160 ng/mL for 2-AG; and 20.5–12,500 ng/mL for AA, respectively. Endogenous and spiked lipids were extracted following the method described above. The quantification was based on the area ratio of analytical standard/internal standards. It was not possible to quantify SEA, due to variability of the results.

Table S1. HCV Peptide Pools Compositions.

	Pool A		
	Core		
	Peptide Length Sequence	Concentration	Volume
1 of 28	18 1 MSTNPKPQRKTKRNTNRR 18	40 mg/mL	25 µL
2 of 28	18 7 PQRKTKRNTNRRPQDVKF 24	40 mg/mL	25 µL
3 of 28	18 14 NTNRRPQDVKFPGGGQIV 31	40 mg/mL	25 µL
4 of 28	17 21 DVKFPGGGQIVGGVYLL 37	40 mg/mL	25 µL
5 of 28	18 27 GGQIVGGVYLLPRRGPRL 44	40 mg/mL	25 µL
6 of 28	18 34 VYLLPRRGPRLGVRATRK 51	40 mg/mL	25 µL
7 of 28	15 41 GPRLGVRATRKTSER 55	40 mg/mL	25 µL
8 of 28	18 45 GVRATRKTSERSQPRGRR 62	40 mg/mL	25 µL

Table S1. *Cont.*

Pool A			
	**Core**		
	**Peptide Length Sequence**	**Concentration**	**Volume**
9 of 28	18 52 TSERSQPRGRRQPIPKAR 69	40 mg/mL	25 µL
10 of 28	18 59 RGRRQPIPKARRPEGRTW 76	40 mg/mL	25 µL
12 of 28	18 73 GRTWAQPGYPWPLYGNEG 90	40 mg/mL	25 µL
13 of 28	18 80 GYPWPLYGNEGCGWAGWL 97	40 mg/mL	25 µL
14 of 28	18 87 GNEGCGWAGWLLSPRGSR 104	40 mg/mL	25 µL
15 of 28	18 94 AGWLLSPRGSRPSWGPTD 111	40 mg/mL	25 µL
16 of 28	17 101 RGSRPSWGPTDPRRRSR 117	40 mg/mL	25 µL
17 of 28	17 107 WGPTDPRRRSRNLGKVI 123	40 mg/mL	25 µL
18 of 28	18 113 RRRSRNLGKVIDTLTCGF 130	40 mg/mL	25 µL
19 of 28	18 120 GKVIDTLTCGFADLMGYI 137	40 mg/mL	25 µL
20 of 28	18 127 TCGFADLMGYIPLVGAPL 144	40 mg/mL	25 µL
21 of 28	18 134 MGYIPLVGAPLGGAARAL 151	40 mg/mL	25 µL
22 of 28	18 141 GAPLGGAARALAHGVRVL 158	40 mg/mL	25 µL
23 of 28	18 148 ARALAHGVRVLEDGVNYA 165	40 mg/mL	25 µL
24 of 28	15 155 VRVLEDGVNYATGNL 169	40 mg/mL	25 µL
25 of 28	18 159 EDGVNYATGNLPGCSFSI 176	40 mg/mL	25 µL
26 of 28	17 166 TGNLPGCSFSIFLLALL 182	40 mg/mL	25 µL
27 of 28	18 172 CSFSIFLLALLSCLTVPA 189	13.3 mg/mL	25 µL
28 of 28	13 179 LALLSCLTVPASA 191	40 mg/mL	25 µL
	**Pool B**		
	**E1**		
	**Peptide Length Sequence**	**Concentration**	**Volume**
1 of 29	18 1 YQVRNSSGLYHVTNDCPN 18	40 mg/mL	20 µL
2 of 29	18 8 GLYHVTNDCPNSSIVYEA 25	40 mg/mL	20 µL
3 of 29	17 15 DCPNSSIVYEAADAILH 31	40 mg/mL	20 µL
4 of 29	16 21 IVYEAADAILHTPGCV 36	40 mg/mL	20 µL
5 of 29	15 26 ADAILHTPGCVPCVR 40	40 mg/mL	20 µL
6 of 29	17 30 LHTPGCVPCVREGNASR 46	40 mg/mL	20 µL
7 of 29	16 36 VPCVREGNASRCWVAV 51	40 mg/mL	20 µL
8 of 29	18 41 EGNASRCWVAVTPTVATR 58	40 mg/mL	20 µL
9 of 29	15 48 WVAVTPTVATRDGKL 62	40 mg/mL	20 µL
10 of 29	18 52 TPTVATRDGKLPTTQLRR 69	40 mg/mL	20 µL
11 of 29	17 59 DGKLPTTQLRRHIDLLV 75	40 mg/mL	20 µL
12 of 29	16 65 TQLRRHIDLLVGSATL 80	40 mg/mL	20 µL
13 of 29	17 70 HIDLLVGSATLCSALYV 86	40 mg/mL	20 µL
14 of 29	18 76 GSATLCSALYVGDLCGSV 93	40 mg/mL	20 µL
15 of 29	18 83 ALYVGDLCGSVFLVGQLF 100	40 mg/mL	20 µL
16 of 29	18 90 CGSVFLVGQLFTFSPRRH 107	40 mg/mL	20 µL
17 of 29	18 97 GQLFTFSPRRHWTTQDCN 114	40 mg/mL	20 µL
18 of 29	18 104 PRRHWTTQDCNCSIYPGH 121	40 mg/mL	20 µL
19 of 29	18 110 TQDCNCSIYPGHITGHRM 127	40 mg/mL	20 µL
20 of 29	17 117 IYPGHITGHRMAWDMMM 133	40 mg/mL	20 µL

Table S1. *Cont.*

	Pool B		
	**E1**		
	**Peptide Length Sequence**	**Concentration**	**Volume**
21 of 29	18 123 TGHRMAWDMMMNWSPTAA 140	40 mg/mL	20 µL
22 of 29	18 130 DMMMNWSPTAALVVAQLL 147	40 mg/mL	20 µL
23 of 29	18 137 PTAALVVAQLLRIPQAIM 154	40 mg/mL	20 µL
24 of 29	18 144 AQLLRIPQAIMDMIAGAH 161	40 mg/mL	20 µL
25 of 29	17 150 PQAIMDMIAGAHWGVLA 166	40 mg/mL	20 µL
26 of 29	18 156 MIAGAHWGVLAGIAYFSM 173	40 mg/mL	20 µL
27 of 29	18 163 GVLAGIAYFSMVGNWAKV 180	40 mg/mL	20 µL
28 of 29	18 170 YFSMVGNWAKVLVVLLLF 187	40 mg/mL	20 µL
29 of 29	16 177 WAKVLVVLLLFAGVDA 192	40 mg/mL	20 µL
	**Pool C**		
	**E2**		
	**Peptide Length Sequence**	**Concentration**	**Volume**
1 of 56	17 1 ETHVTGGNAGRTTAGLV 17	40 mg/mL	25 µL
2 of 56	18 7 GNAGRTTAGLVGLLTPGA 24	40 mg/mL	25 µL
3 of 56	18 14 AGLVGLLTPGAKQNIQLI 31	40 mg/mL	25 µL
4 of 56	18 21 TPGAKQNIQLINTNGSWH 38	40 mg/mL	25 µL
5 of 56	17 28 IQLINTNGSWHINSTAL 44	40 mg/mL	25 µL
6 of 56	17 34 NGSWHINSTALNCNESL 50	40 mg/mL	25 µL
7 of 56	17 40 NSTALNCNESLNTGWLA 56	40mg/mL	25 µL
8 of 56	18 46 CNESLNTGWLAGLFYQHK 63	40 mg/mL	25 µL
9 of 56	18 53 GWLAGLFYQHKFNSSGCP 70	40 mg/mL	25 µL
10 of 56	18 60 YQHKFNSSGCPERLASCR 77	40 mg/mL	25 µL
11 of 56	17 67 SGCPERLASCRRLTDFA 83	40 mg/mL	25 µL
12 of 56	17 73 LASCRRLTDFAQGWGPI 89	40 mg/mL	25 µL
13 of 56	19 79 LTDFAQGWGPISYANGSGL 97	40 mg/mL	25 µL
14 of 56	18 87 GPISYANGSGLDERPYCW 104	40mg/mL	25 µL
15 of 56	16 94 GSGLDERPYCWHYPPR 109	40 mg/mL	25 µL
16 of 56	18 99 ERPYCWHYPPRPCGIVPA 116	40 mg/mL	25 µL
17 of 56	18 106 YPPRPCGIVPAKSVCGPV 123	40 mg/mL	25 µL
18 of 56	19 113 IVPAKSVCGPVYCFTPSPV 131	40 mg/mL	25 µL
19 of 56	18 121 GPVYCFTPSPVVVGTTDR 138	40 mg/mL	25 µL
20 of 56	17 128 PSPVVVGTTDRSGAPTY 144	40 mg/mL	25 µL
21 of 56	15 134 GTTDRSGAPTYSWGA 148	40 mg/mL	25 µL
22 of 56	18 138 RSGAPTYSWGANDTDVFV 155	40 mg/mL	25 µL
23 of 56	16 145 SWGANDTDVFVLNNTR 160	40 mg/mL	25 µL
24 of 56	18 150 DTDVFVLNNTRPPLGNWF 167	40 mg/mL	25 µL
25 of 56	16 157 NNTRPPLGNWFGCTWM 172	40 mg/mL	25 µL
26 of 56	18 162 PLGNWFGCTWMNSTGFTK 179	40 mg/mL	25 µL
27 of 56	15 169 CTWMNSTGFTKVCGA 183	40 mg/mL	25 µL
28 of 56	16 173 NSTGFTKVCGAPPCVI 188	40 mg/mL	25 µL
29 of 56	19 178 TKVCGAPPCVIGGVGNNTL 196	40 mg/mL	25 µL
30 of 56	18 186 CVIGGVGNNTLLCPTDCF 203	40 mg/mL	25 µL

Table S1. *Cont.*

Peptide Length Sequence	Concentration	Volume
**Pool C**		
**E2**		
**Peptide Length Sequence**	**Concentration**	**Volume**
31 of 56 17 193 NNTLLCPTDCFRKHPEA 209	40 mg/mL	25 µL
32 of 56 15 199 PTDCFRKHPEATYSR 213	40 mg/mL	25 µL
33 of 56 18 203 FRKHPEATYSRCGSGPWI 220	40 mg/mL	25 µL
34 of 56 17 210 TYSRCGSGPWITPRCMV 226	40 mg/mL	25 µL
35 of 56 18 216 SGPWITPRCMVDYPYRLW 233	40 mg/mL	25 µL
36 of 56 17 223 RCMVDYPYRLWHYPCTI 239	40 mg/mL	25 µL
37 of 56 18 229 PYRLWHYPCTINYTIFKV 246	40 mg/mL	25 µL
38 of 56 18 236 PCTINYTIFKVRMYVGGV 253	40 mg/mL	25 µL
39 of 56 18 243 IFKVRMYVGGVEHRLEAA 260	40 mg/mL	25 µL
40 of 56 16 250 VGGVEHRLEAACNWTR 265	40 mg/mL	25 µL
41 of 56 17 255 HRLEAACNWTRGERCDL 271	40 mg/mL	25 µL
42 of 56 16 261 CNWTRGERCDLEDRDR 276	40 mg/mL	25 µL
43 of 56 18 266 GERCDLEDRDRSELSPLL 283	40 mg/mL	25 µL
44 of 56 17 273 DRDRSELSPLLLSTTQW 289	40 mg/mL	25 µL
45 of 56 18 279 LSPLLLSTTQWQVLPCSF 296	40 mg/mL	25 µL
46 of 56 17 286 TTQWQVLPCSFTTLPAL 302	40 mg/mL	25 µL
47 of 56 18 292 LPCSFTTLPALSTGLIHL 309	40 mg/mL	25 µL
48 of 56 18 299 LPALSTGLIHLHQNIVDV 316	40 mg/mL	25 µL
49 of 56 17 306 LIHLHQNIVDVQYLYGV 322	40 mg/mL	25 µL
50 of 56 18 312 NIVDVQYLYGVGSSIASW 329	40 mg/mL	25 µL
51 of 56 18 319 LYGVGSSIASWAIKWEYV 336	40 mg/mL	25 µL
52 of 56 18 326 IASWAIKWEYVVLLFLLL 343	40 mg/mL	25 µL
53 of 56 16 333 WEYVVLLFLLLADARV 348	20 mg/mL	25 µL
54 of 56 18 338 LLFLLLADARVCSCLWMM 355	40 mg/mL	25 µL
55 of 56 17 345 DARVCSCLWMMLLISQA 361	40 mg/mL	25 µL
56 of 56 13 351 CLWMMLLISQAEA 363	40 mg/mL	25 µL
**Pool D**		
**NS2**		
**Peptide Length Sequence**	**Concentration**	**Volume**
1 of 32 18 1 LDTEVAASCGGVVLVGLM 18	20 mg/mL	50 µL
2 of 32 18 8 SCGGVVLVGLMALTLSPY 25	20 mg/mL	50 µL
3 of 32 18 15 VGLMALTLSPYYKRYISW 32	40 mg/mL	25 µL
4 of 32 18 22 LSPYYKRYISWCMWWLQY 39	40 mg/mL	25 µL
5 of 32 18 29 YISWCMWWLQYFLTRVEA 46	40 mg/mL	25 µL
6 of 32 17 36 WLQYFLTRVEAQLHVWV 52	40 mg/mL	25 µL
7 of 32 17 42 TRVEAQLHVWVPPLNVR 58	40mg/mL	25 µL
8 of 32 18 48 LHVWVPPLNVRGGRDAVI 65	40mg/mL	25 µL
9 of 32 18 55 LNVRGGRDAVILLMCVVH 72	40mg/mL	25 µL
10 of 32 18 62 DAVILLMCVVHPTLVFDI 79	40mg/mL	25 µL
11 of 32 18 69 CVVHPTLVFDITKLLLAI 86	40 mg/mL	25 µL
12 of 32 18 76 VFDITKLLLAIFGPLWIL 93	40 mg/mL	25 µL
13 of 32 18 83 LLAIFGPLWILQASLLKV 100	40 mg/mL	25 µL

Table S1. *Cont.*

Peptide	Length Sequence	Concentration	Volume
**Pool D**			
**NS2**			
**Peptide**	**Length Sequence**	**Concentration**	**Volume**
14 of 32	17 90 LWILQASLLKVPYFVRV 106	40 mg/mL	25 µL
15 of 32	17 96 SLLKVPYFVRVQGLLRI 112	40 mg/mL	25 µL
16 of 32	18 102 YFVRVQGLLRICALARKI 119	40 mg/mL	25 µL
17 of 32	17 109 LLRICALARKIAGGHYV 125	40 mg/mL	25 µL
18 of 32	18 115 LARKIAGGHYVQMAIIKL 132	40 mg/mL	25 µL
19 of 32	18 122 GHYVQMAIIKLGALTGTY 139	40 mg/mL	25 µL
20 of 32	16 129 IIKLGALTGTYVYNHL 144	40 mg/mL	25 µL
21 of 32	18 134 ALTGTYVYNHLTPLRDWA 151	40 mg/mL	25 µL
22 of 32	18 141 YNHLTPLRDWAHNGLRDL 158	40 mg/mL	25 µL
23 of 32	18 148 RDWAHNGLRDLAVAVEPV 165	40 mg/mL	25 µL
24 of 32	16 155 LRDLAVAVEPVVFSRM 170	40 mg/mL	25 µL
25 of 32	18 160 VAVEPVVFSRMETKLITW 177	40 mg/mL	25 µL
26 of 32	17 167 FSRMETKLITWGADTAA 183	40 mg/mL	25 µL
27 of 32	16 173 KLITWGADTAACGDII 188	40 mg/mL	25 µL
28 of 32	18 178 GADTAACGDIINGLPVSA 195	40 mg/mL	25 µL
29 of 32	18 185 GDIINGLPVSARRGQEIL 202	40 mg/mL	25 µL
30 of 32	18 192 PVSARRGQEILLGPADGM 209	40 mg/mL	25 µL
31 of 32	18 198 GQEILLGPADGMVSKGWR 215	40 mg/mL	25 µL
32 of 32	13 205 PADGMVSKGWRLL 217	40 mg/mL	25 µL
**Pool E**			
**NS3-1**			
**Peptide**	**Length Sequence**		
1 of 98	18 1 APITAYAQQTRGLLGCII 18		
2 of 98	18 7 AQQTRGLLGCIITSLTGR 24		
3 of 98	16 14 LGCIITSLTGRDKNQV 29		
4 of 98	18 19 TSLTGRDKNQVEGEVQIV 36		
5 of 98	18 26 KNQVEGEVQIVSTATQTF 43		
6 of 98	16 33 VQIVSTATQTFLATCI 48		
7 of 98	18 38 TATQTFLATCINGVCWTV 55		
8 of 98	18 45 ATCINGVCWTVYHGAGTR 62		
9 of 98	17 52 CWTVYHGAGTRTIASPK 68		
10 of 98	18 58 GAGTRTIASPKGPVIQMY 75		
11 of 98	18 65 ASPKGPVIQMYTNVDQDL 82		
12 of 98	16 72 IQMYTNVDQDLVGWPA 87		
13 of 98	18 77 NVDQDLVGWPAPQGSRSL 94		
14 of 98	18 84 GWPAPQGSRSLTPCTCGS 101		
15 of 98	17 91 SRSLTPCTCGSSDLYLV 107		
16 of 98	18 97 CTCGSSDLYLVTRHADVI 114		
17 of 98	16 104 LYLVTRHADVIPVRRR 119		
18 of 98	18 109 RHADVIPVRRRGDSRGSL 126		
19 of 98	17 116 VRRRGDSRGSLLSPRPI 132		
20 of 98	15 122 SRGSLLSPRPISYLK 136		

Table S1. *Cont.*

Peptide	Length	Sequence
**Pool E**		
**NS3-1**		
**Peptide**	**Length**	**Sequence**
21 of 98	18	126 LLSPRPISYLKGSSGGPL 143
22 of 98	18	133 SYLKGSSGGPLLCPAGHA 150
23 of 98	18	140 GGPLLCPAGHAVGLFRAA 157
24 of 98	18	147 AGHAVGLFRAAVCTRGVA 164
25 of 98	17	154 FRAAVCTRGVAKAVDFI 170
26 of 98	16	160 TRGVAKAVDFIPVENL 175
27 of 98	16	165 KAVDFIPVENLGTTMR 180
28 of 98	15	170 IPVENLGTTMRSPVF 184
29 of 98	19	174 NLGTTMRSPVFTDNSSPPA 192
30 of 98	18	182 PVFTDNSSPPAVPQSFQV 199
31 of 98	16	189 SPPAVPQSFQVAHLHA 204
32 of 98	17	194 PQSFQVAHLHAPTGSGK 210
33 of 98	18	200 AHLHAPTGSGKSTKVPAA 217
**Pool E**		
**NS3-2**		
**Peptide**	**Length**	**Sequence**
34 of 98	18	207 GSGKSTKVPAAYAAQGYK 224
35 of 98	15	214 VPAAYAAQGYKVLVL 228
36 of 98	17	218 YAAQGYKVLVLNPSVAA 234
37 of 98	18	224 KVLVLNPSVAATLGFGAY 241
38 of 98	18	231 SVAATLGFGAYMSKAHGV 248
39 of 98	16	238 FGAYMSKAHGVDPNIR 253
40 of 98	17	243 SKAHGVDPNIRTGVRTI 259
41 of 98	17	249 DPNIRTGVRTITTGSPI 265
42 of 98	18	255 GVRTITTGSPITYSTYGK 272
43 of 98	18	262 GSPITYSTYGKFLADGGC 279
44 of 98	18	269 TYGKFLADGGCSGGAYDI 286
45 of 98	18	276 DGGCSGGAYDIIICDECH 293
46 of 98	18	283 AYDIIICDECHSTDATSI 300
47 of 98	18	290 DECHSTDATSILGIGTVL 307
48 of 98	17	297 ATSILGIGTVLDQAETA 313
49 of 98	18	303 IGTVLDQAETAGARLVVL 320
50 of 98	18	310 AETAGARLVVLATATPPG 327
51 of 98	17	317 LVVLATATPPGSVTVSH 333
52 of 98	18	323 ATPPGSVTVSHPNIEEVA 340
53 of 98	18	330 TVSHPNIEEVALSTTGEI 347
54 of 98	18	337 EEVALSTTGEIPFYGKAI 354
55 of 98	17	344 TGEIPFYGKAIPLEVIK 360
56 of 98	18	350 YGKAIPLEVIKGGRHLIF 367
57 of 98	17	357 EVIKGGRHLIFCHSKKK 373
58 of 98	18	363 RHLIFCHSKKKCDELAAK 380

Table S1. *Cont.*

Pool E				
NS3-2				
Peptide	Length	Sequence		
59 of 98	17	370	SKKKCDELAAKLVALGI	386
60 of 98	18	376	ELAAKLVALGINAVAYYR	393
61 of 98	18	383	ALGINAVAYYRGLDVSVI	400
62 of 98	18	390	AYYRGLDVSVIPTSGDVV	407
63 of 98	18	397	VSVIPTSGDVVVVSTDAL	414
64 of 98	15	404	GDVVVVSTDALMTGF	418
65 of 98	18	408	VVSTDALMTGFTGDFDSV	425
